# Long-stay in forensic-psychiatric care in the UK

**DOI:** 10.1007/s00127-017-1473-y

**Published:** 2018-01-31

**Authors:** Laurie Hare Duke, Vivek Furtado, Boliang Guo, Birgit Angela Völlm

**Affiliations:** 1 Division of Psychiatry and Applied Psychology, School of Medicine, Institute of Mental Health, University of Nottingham, Jubilee Campus, Triumph Road, Nottingham, NG7 2TU UK; 2 University of Warwick, Coventry, UK; 3 Nottinghamshire Healthcare NHS Foundation Trust, Nottingham, UK

**Keywords:** Forensic, Secure setting, Mentally disordered offender, Length of stay, Characteristics

## Abstract

**Purpose:**

Forensic services provide care for mentally disordered offenders. In England this is provided at three levels of security—low, medium and high. Significant number of patients within these settings remain detained for protracted periods of time. This is both very costly and restrictive for individuals. No national studies have been conducted on this subject in England.

**Methods:**

We employed a cross-sectional design using anonymised data from medical records departments in English secure forensic units. Data were collected from a large sample of medium secure patients (*n* = 1572) as well as the total high secure patient population (*n* = 715) resident on the census date (01-04-2013). We defined long-stay as a stay of more than 10 years in high, 5 years in medium or 15 years in a mix of high and medium secure settings. Long-stay status was assessed against patient demographic and admission information.

**Results:**

We identified a significant proportion of long-stayers: 23.5% in high secure and 18.1% in medium secure care. Amongst medium secure units a large variation in long-stay prevalence was observed from 0 to 50%. Results indicated that MHA section, admission source and current ward type were independent factors associated with long-stay status.

**Conclusion:**

This study identified a significant proportion of long-stayers in forensic settings in England. Sociodemographic factors identified in studies in individual settings may be less important than previously thought. The large variation in prevalence of long-stayers observed in the medium secure sample warrants further investigation.

## Background

The purpose of forensic-psychiatric care is to improve the mental health of mentally disordered offenders whilst reducing their risk of recidivism. Forensic-psychiatric services in England provide care and treatment for mentally disordered offenders in high, medium and low secure in-patient facilities as well as in the community. High secure units (HSUs) admit individuals detained under the Mental Health Act and who “require treatment under conditions of high security on account of their dangerous, violent or criminal propensities” [1]. Medium secure units (MSUs) were developed in the late 1970s to bridge the gap between high secure and general psychiatric care and are designed for those patients detained under the Mental Health Act who “pose a serious danger to the public” [1]. There are currently three high secure hospitals in England providing just over 700 beds and around 60 medium secure units providing around 3500 medium secure beds, with nearly 35% of those beds provided by the independent sector.

Since the 1950s there has been an increasing tendency towards deinstitutionalisation, with more patients being treated in community settings rather than as inpatients on general psychiatric hospitals. This process has been consistently associated with greater user satisfaction, increased met needs, and better outcomes on adherence to treatment, clinical symptoms and quality of life [2–5]. Whilst bed numbers have decreased in general psychiatric hospitals, they have actually increased in forensic-psychiatric services over the same period.

A significant number of patients in secure units remain in care for extended periods of time. In addition, it has previously been found that between one and two-thirds of patients in high secure settings in the UK did not need that level of security [6–10]. Meanwhile it appears that the average length of stay (LoS) within MSUs is on the rise [11] and it is similarly expected that a substantial proportion of patients will be placed under restrictions inappropriate to their level of risk.

There are strong ethical and financial concerns arising from potentially unnecessarily protracted stay in secure care. Secure settings are extremely restrictive, characterised by a loss of privacy, repetitive daily routines and low-stimulation environments. Although this may be necessary for some patients, it is of concern that some individuals remain in secure care for potentially inappropriate lengths of time. Secure care provision is also very expensive. MSUs in the UK, for example, cost around £175,000 per annum per patient, consuming £1.2 billion per annum. This is 1% of the entire NHS and 10% of the mental health budget [1, 12]. Services must therefore aim to target only those individuals who require and will benefit from them.

At the same time, there is a suggestion that community based services do not provide sufficient levels of care for a sub-group of forensic patients, for whom ‘deinstitutionalisation’ may not be appropriate [13]. There has been some recognition of this group of patients at the international level. A review of the international literature revealed two European countries who have responded proactively to the needs of those patients who require long-term forensic-psychiatric care. In both the Netherlands and Germany long-stay units have been developed. It has been found that purposefully designed long-stay wards in the Netherlands may attract some cost savings compared to regular treatment wards as well as increased patient satisfaction due to their focus on quality of life [14]. Thus, identifying the characteristics of long-stay patients can support service improvements not only in order to better facilitate patient discharge, but also to aid in the development of more cost-effective pathways with better quality of life for patients genuinely requiring longer term care.

It is imperative therefore to identify the characteristics of long-stay patients and the factors behind their LoS in order to design appropriate service models to meet their particular needs. Previous studies in secure settings have identified a number of predictors of LoS: severity of index offence, psychopathology, referral from another secure or psychiatric setting, restriction orders and lack of facilities with lower levels of care and security [11, 15, 16]. However, these previous studies have been based upon samples from single units and no national studies are currently available on long-stay in forensic-psychiatric care in England. This may hinder the provision of services to support the discharge or to improve their quality of life within secure care of this patient group [17, 18].

### Aims and objectives

Using a cross-sectional approach, this study aimed to: (1) identify the prevalence of long-stay in high secure units in England; (2) estimate the prevalence of long-stay in medium secure units in England and (3) identify individual-level sociodemographic and service factors associated with long-stay amongst patients in high and medium secure care in England.

## Method

### Design and approval

A cross-sectional design was used, collecting anonymised data from all high secure units as well as a sample of medium secure units in England resident on the census date (01-04-2013). Individuals who were on trial leave at the time were excluded. Data were submitted to the research team in anonymised form and only included routinely collected data. As such the study did not require ethical approval and was deemed to fall within the remit of service evaluation by the sponsoring organisation.

### Definition of ‘long-stay’

Patients were categorised into long-stayers and non-long-stay patients. Our piloting data from one high secure care setting suggested that just over 15% of patients stayed for over 10 years. For medium secure care, the literature suggests that 10–20% stay 5 years or longer. For our study, we aimed to capture the extreme end of long-stay; therefore, a cut-off that would capture around 15–20% of the population seemed appropriate. This is also the percentage of patients in long-stay services in countries where dedicated long-stay services exist. Allocation to ‘long-stay’ status was determined by total time of *continuous* stay in high and/or medium secure care, i.e. from admission to any such setting to census date. Long-stay was defined as five or more continuous years in medium secure care OR ten or more continuous years in high secure care OR a combination of the high and medium secure settings totalling 15 years or more of continuous secure care. Assignment to long-stay status was initially by review of admission date to current unit though if patients did not fall within the long-stay category based on their LoS in the current unit, we then enquired about admission source and, if admission source was high or medium secure care, whether the individual fulfils our criteria for long-stay through medical records staff or responsible clinicians. Unfortunately, date of first admission to secure care was not readily available in all cases, i.e. clinicians would be able to say with confidence that a particular individuals had been in services in excess of our cut-off but without being able to identify exact LoS overall in secure care; this is the reason why we were not able to use LoS as a continuous variable in the analyses.

### Selection of participating units

All three high secure units in England were included.

A stratified cluster sampling frame was adopted for MSUs with 23 units sampled. This included 14 NHS and 9 independent units, drawn according to geographical region (according to boundaries of the then 10 Strategic Health Authorities), size and specialisation, with oversampling of units specialising in particular patient groups (e.g. patients with intellectual disabilities). This sample represents approximately 40% of all MSUs in England. One medium secure unit was included in regions with 1 to 3 units, 2 in regions with 4 or 5 units, 3 in regions with 6 or 7 units, 4 in regions with 8 or 9 units and 5 in regions with 10 or more medium secure units. Based on patient numbers, 11 (48%) of these units were classed as small (≤ 50 patients), 7 (30%) were medium-sized (51–99 patients) and 5 (22%) were large (≥ 100 patients). Units were located across all English regions: North East (*n* = 1), North West (4), Yorkshire and the Humber (2), East Midlands (2), West Midlands (2), East of England (4), London (3), South East (2), South Central (1), South West (2).

### Data collected

Data for both HSUs and MSUs were collected through medical records departments on length of stay and basic patient and admission characteristics. Data collected were based on information known to be readily available from administrative systems on the basis of a pilot trial conducted in one HSU and one MSU. This included the following variables: date of admission to current unit, age, gender, ethnic class (White, Black, Asian, mixed, other), admission source, current Mental Health Act (MHA) section; diagnostic specification of current ward [mental illness, personality disorder (PD), comorbidity, intellectual disability (ID), neuropsychiatry, mixed diagnosis, other, cannot assign] and stage of treatment specification of current ward (admission/assessment, treatment, high dependency, long-stay/slow stream, pre-discharge/rehab, mixed assessment/treatment, other, cannot assign).

### Data analysis

Admission source was collapsed into community (any non-secure psychiatric settings, including Psychiatric Intensive Care Units, non-institutional settings, police stations), low, medium and high secure settings and prison. MHA section was categorised as civil/quasi-civil (s2, 3, 37, 37(N), 41(5), 47), hospital orders with restriction (s37/41, CPIA), prison transfer (s47/49, 48/49), pre-sentencing (s35, 36, 38) and other.^1^

Data analysis was conducted separately for patients in high and medium secure settings as factors determining length of stay might be different in both settings, and only the former constitutes the full population. Results presented for the medium secure analysis are adjusted for sampling weights. Summary statistics were taken of all included variables.

National level information on the demographics and service use variables used in the sampling stratification is currently unavailable, which precluded adjustment for unequal probability of selection within the medium secure sample. The variability of long-stay status across units was first investigated in a two-level logistic regression with the secure unit as the level two analytical unit. Results showed no significant unit-level variability amongst HSUs, but statistically significant variability among MSUs (var = 0.491, 95% CI 0.186–1.292, intra-class correlation: 13.0%). Exploratory analysis further showed non-significant region-level variance among units. Therefore, a general logistic regression was used to explore the association between long-stay and various influential factors for the high secure sample and a two-level logistic regression was used for the medium secure sample. As exploratory analysis showed there were some missing values for some influential factors (ethnic class: 7.4%, admission source: 9.1%), the robustness of the results was assessed by comparing the results of modelling data with missing covariates imputed by means of multiple imputation. All analysis was conducted using STATA 15.

For the high secure sample, univariate analysis was conducted with each factor entered individually into a logistic regression model to allow comparison of associations with and without adjusting other factors. For the medium secure sample a series of two-level logistic regressions were run, with the secure unit entered as the level 2 analytical unit. All factors were subsequently entered simultaneously into a multivariate model.

As this study is exploratory, no hypotheses were made regarding the analysis.

## Results

### Prevalence of long-stay

According to our criteria, the prevalence of long-stay in across all three English high secure settings was 23.5%, ranging from 21.6 to 26.5%. Within the medium secure sample, the prevalence of long-stay was 18.1%. There was a wide degree of variation between the medium secure units in our sample, with long-stay prevalence ranging from 0 to 50%. With sampling weights and adjustment for unit-level variance, the predicted probability for long-stay in the medium secure sample was 16.9% (95% CI 12.7%, 21.1%) (Table 1).

### Factors associated with long-stay status

Variables entered in logistic models included MHA section and admission source; for the high secure sample ward diagnostic category was entered additionally and for the medium secure sample ward pathway category. For the high secure analysis the categories ‘low secure unit’ from admission source and ‘pre-sentencing’ and ‘other’ from the MHA section variable were omitted given inadequate number of long-stay cases; the latter two categories were also omitted from the medium secure analysis.

#### High secure care

Results for the high secure population are shown in Table 2. The multivariate analysis found that demographic variables (gender and ethnic class) were not significantly associated with long-stay. For ethnic class, additional analysis of white patients compared to non-white was also non-significant (not shown in table). Compared with patients admitted on s37/41, other MHA section types were associated with a significantly reduced likelihood of long-stay status by over half. Those with a civil/quasi-civil section had 42% reduced odds and patients on a prison transfer had 68% reduced odds. Admission source demonstrated a significantly increased likelihood of long-stay against prison admissions for previous high secure cases and community admissions with medium secure admissions being non-significant (OR = 1.257, *p* = .369). It should be noted that there were only 5 cases admitted from the community in the high secure sample and estimates may not be reliable for this group. Diagnostic ward categorisation was a significant factor when comparing intellectual disability against personality disorder wards with cases from the latter presenting with reduced likelihood of prolonged stay. It may also be noted that patients on intellectual disability ward were also more likely to be long-stayers compared to mixed type and mental illness wards at the marginal significance level (*p* = .081 and 0.076, respectively, not shown in Table 2).

**Table 1:** Frequencies of patient, pathway and MHA section factors in the high and medium secure samples

Patient, pathway and MHA section factors	High security		Medium security	
	Long-stay patients (*n* = 168)	Non-long-stay patients (*n* = 547)	Long-stay patients (*n* = 285)	Non-long-stay patients (*n* = 1287)
Provider, *n* (%)				
NHS			178 (62)	915 (71)
Independent			107 (38)	372 (29)
LoS in current unit (days), median (P25, P75)	4294 (2088, 5844)	1332 (628, 2161)	1560 (605, 2383)	438 (168, 838)
LoS in current unit (months), median (P25, P75)	141 (69, 192)	44 (21, 71)	51 (20, 78)	14 (6, 28)
Age, mean (SD)	45.43 (9.67)	36.15 (9.72)	43.87 (11.74)	34.68 (11.21)
Gender, *n* (%)				
Male	157 (93.5)	514 (94.0)	240 (84.2)	1049 (81.5)
Female	11 (6.5)	33 (6.0)	45 (15.8)	238 (18.8)
Ethnic class, *n* (%)				
White	128 (76.7)	404 (75.0)	216 (77.8)	808 (68.6)
Black	23 (13.8)	75 (13.9)	39 (14.1)	208 (17.6)
Asian	9 (5.4)	22 (4.1)	9 (3.2)	81 (6.9)
Mixed	6 (3.6)	32 (5.9)	12 (4.4)	61 (5.2)
Other	1 (< 1)	6 (1.1)	1 (< 1)	20 (1.7)
Region, *n* (%)				
East Midlands			27 (9.4)	208 (16.1)
East of England			50 (17.5)	144 (11.1)
North East			19 (6.6)	58 (4.5)
North West			60 (21.0)	217 (16.8)
Yorkshire			48 (16.8)	108 (8.3)
West Midlands			6 (2.1)	134 (10.4)
London			46 (16.1)	254 (19.7)
South East			7 (2.4)	54 (4.2)
South West			16 (5.6)	81 (6.2)
South Central			6 (2.1)	29 (2.2)
MHA section, *n* (%)				
Civil/quasi-civil	30 (17.9)	111 (20.3)	81 (28.2)	492 (38.2)
Hospital order with restrictions	108 (64.3)	205 (37.5)	185 (64.9)	516 (40.1)
Prison transfer	30 (17.9)	227 (41.5)	19 (6.7)	242 (18.8)
Pre-sentencing	0	4 (0.7)	0	17 (1.3)
Other	0	0	0	20 (1.6)
Admission source, *n* (%)				
Community	4 (2.5)	1 (< 1)	12 (4.7)	179 (15.3)
Prison	65 (41.1)	367 (70.3)	46 (18.0)	602 (51.3)
Low secure unit	0	2 (< 1)	8 (3.1)	119 (10.1)
Medium secure unit	51 (32.2)	137 (26.2)	118 (46.1)	192 (16.4)
High secure unit	38 (24.1)	15 (2.9)	72 (28.1)	81 (6.9)
Ward diagnostic category, *n* (%)				
Mental illness	78 (46.4)	245 (44.8)	132 (46.3)	472 (36.7)
Personality disorder	48 (28.6)	209 (38.2)	20 (7.0)	80 (6.2)
Learning difficulties	24 (14.3)	34 (6.2)	31 (10.9)	143 (11.1)
Mixed/other	18 (10.7)	59 (10.9)	102 (35.8)	592 (46.0)
Ward pathway category, *n* (%)				
Admission	8 (4.8)	46 (8.4)	9 (3.2)	201 (15.6)
Treatment	87 (51.8)	294 (53.7)	83 (20.1)	382 (29.7)
High dependency	26 (15.5)	72 (13.2)	1 (< 1)	28 (2.2)
Slow/rehab	19 (11.3)	61 (11.2)	101 (35.4)	221 (17.2)
Mixed/other	28 (16.7)	74 (13.5)	91 (31.9)	455 (35.4)

**Table 2:** Long-stay status and associated factors in high secure care

Parameters	Bivariate, odds ratio (SE)	Multivariate, odds ratio (SE)
Gender		
Female	1.000	
Male	0.916 (0.329)	1.072 (0.548)
Ethnic class		
White	1.000	
Black	0.967 (0.250)	0.577 (0.191)
Asian	1.291 (0.527)	1.095 (0.492)
Mixed	0.591 (0.270)	0.517 (0.267)
Other	0.526 (0.570)	0.266 (0.336)
MHA		
Hospital order w/restrictions	1.000	1.000
Civil/ quasi-civil	0.513 (0.121)**	0.579 (0.158)*
Prison transfer	0.250 (0.057)***	0.328 (0.088)***
Admission source		
Prison	1.000	1.000
Community	22.584 (25.432)**	15.379 (18.134)*
Medium secure	2.101 (0.445)***	1.257 (0.320)
High secure	14.303 (4.767)***	11.235 (3.986)***
Ward pathway category		
Mixed/other	1.000	
Admission	0.459 (0.203)	0.476 (0.265)
Treatment	0.782 (0.198)	0.926 (0.288)
High dependency	0.954 (0.304)	0.755 (0.292)
Slow/rehab	0.823 (0.283)	0.680 (0.277)
Ward diagnostic category		
Intellectual disability	1.000	1.000
Mixed/other	0.432 (0.163)*	0.445 (0.206)
Personality disorder	0.325 (0.101)***	0.422 (0.159)*
Mental illness	0.451 (0.133)**	0.530 (0.189)

* *p* < .05, ** *p* < .01, *** *p* < .001

#### Medium secure care

Similarly to the high secure population, the multivariate results for the medium secure sample in Table 3 show that demographic variables (gender and ethnic class) were not significantly associated with long-stay. For ethnic class, additional analysis of white patients compared to non-white was also non-significant (not shown in table). Regarding MHA section, compared with patients sectioned on hospital orders with restrictions, those on civil/quasi-civil section had 63% reduced odds of being a long-stayer and the odds were reduced by 65% for prison transfer patients. Admission source was also a significant factor. Compared to prison-admitted patients, cases arriving from medium and high secure settings had approximately eight times the odds of being a long-stayer. For ward diagnostic category, patients in learning disability wards showed increased odds of long-stay compared to all other ward types, though none of the results reached significance level.

**Table 3:** Long-stay status and associated factors in medium secure care

Parameters	Bivariate, odds ratio (SE)	Multivariate, odds ratio (SE)
Gender		
Female	1.000	
Male	0.927 (0.212)	0.744 (0.281)
Ethnic class		
White	1.000	
Black	0.945 (0.191)	0.962 (0.313)
Asian	0.808 (0.263)	1.049 (0.516)
Mixed	0.916 (0.391)	0.814 (0.288)
Other	0.335 (0.239)	0.571 (0.486)
Provider		
NHS	1.000	
Independent	1.376 (0.516)	1.072 (0.303)
MHA		
Hospital order w/restrictions	1.000	
Civil/ quasi-civil	0.339 (0.080)***	0.365 (0.106)***
Prison transfer	0.231 (0.087)***	0.347 (0.099)***
Admission source		
Prison	1.000	
Community	0.521 (0.206)	0.518 (0.209)
Low secure	0.526 (0.278)	7.960 (1.751)
Medium secure	8.106 (2.621)***	0.644 (0.319)***
High secure	8.347 (2.285)***	8.087 (2.316)***
Ward pathway category		
Mixed/other	1.000	
Admission	0.362 (0.238)	0.733 (0.432)
Treatment	0.914 (0.263)	1.122 (0.450)
High dependency	0.307 (0.197)	1.569 (1.801)
Slow/rehab	1.468 (0.946)	1.784 (1.231)
Ward diagnostic category		
Intellectual disability	1.000	
Mixed/other	0.757 (0.258)	0.544 (0.180)
Personality disorder	0.863 (0.298)	0.533 (0.312)
Mental illness	1.075 (0.281)	0.790 (0.360)

* *p* < .05, ** *p* < .01, *** *p* < .001

## Discussion

This study sought to assess the prevalence of long-stay as well as some of the key determinants of long-stay status within high and medium secure care settings in England. According to our specified criteria, 24% of patients within high secure units were classified as long-stayers and an estimated 17.4% in medium secure. There is limited research identifying how many patients stay for extended periods of time in high or medium secure hospitals in England and comparisons with previous studies are difficult to draw as previous research has used different cut-offs for ‘long-stay’, calculated LoS in patients’ current unit only rather than continuous care, and sampled from single units.

In high secure care, Dell et al. [16] found that 44.4% of patients had exceeded the average LoS of 8 years in their study at one high secure hospital. This would appear to be a higher figure than ours though their study used a lower LoS cut-off; in addition, the data of that study is 20 years old now and policy and pathways have changed - not least has the accelerated discharge programme since taken place [19] targeting some of the residents in the Dell et al. [16] study.

Meanwhile in medium secure settings studies using our cut-off of 5 years for LoS in England reported figures of between just under 10 to just over 20% [20–22]. Two of these figures are lower than ours but these used current unit LoS and sampled from single units. Given the huge variation in prevalence between both units in our study, it is clear that research in one single setting does not provide a useful national picture of LoS.

The large variation in prevalence of long-stay for medium secure care is worth noting. One of the units included here had a ward set up specifically for those leaving high secure care as part of the accelerated discharge programme—therefore, a higher percentage of long-stayers in this unit was expected. On the other hand, about two-thirds of the high and half of the medium secure long-stay group were admitted from the same or lower levels of security. Variation in long-stay numbers may arise as a result of the different patient groups (e.g. those with PD or LD) catered for; some studies have also identified variation in admission rates by geographical location due to differences in social deprivation, ethnic class and availability of low secure beds [23]. These factors are unlikely to fully account for the differences in long-stay though, particularly as we did not find some of them, e.g. ethnic class, to be associated with long-stay status. There are no national standards with regard to admission criteria to medium secure care beyond the patient being a “serious danger to the public” [1] and it is possible, though this cannot be confirmed by our study, that individual units adopt their own (implicit or explicit) criteria, such as not admitting patients with little prospect of moving on to less secure settings or being discharged. Alternatively, it is possible that the interventions offered in units with a higher proportion of long-stayers are less effective in allowing patients to move on.

Our final models suggested that MHA section, admission source and current ward type were each independently associated with long-stay status. Previous studies have produced somewhat conflicting findings with regard to associations between sociodemographic factors and LoS though most have not found such a relationship. Two previous studies have identified that non-white patients had a shorter LoS than white ethnic groups [21, 22] and studies that looked at gender differences have found shorter LoS in females [24]. Notably, their longer term outcome seems to be worse though [25]. We did not find any difference between long-stayers and non-long-stayers on gender or ethnic class; the higher percentage of white ethnic class in long-stayers in the medium secure setting failed to reach statistical significance. Unsurprisingly, long-stayers were older than non-long-stayers in both, high and medium secure care. The large number of older patients, with about one-third of the long-stay population being over 50, has important implications for the service planning for this patient group.

In line with other research in individual settings [15, 24, 26] our national study has also identified an association between MHA and long-stay status in both, medium and high secure patients with significantly more patients in the long-stay groups on hospital orders with restrictions and less on prison transfers. This reflects the practical realities of this section in that it does not allow return transfer back to prison for those who may no longer benefit from hospital treatment. Compared to those civil sections (or quasi-civil sections, such as hospital orders without restrictions) these patients also require Ministry of Justice approval for moves to other secure settings, another reason for the potential delay in their transfer. The data on admission source additionally reflects potential challenges in the smooth transfer of this patient group along a pathway from more to less secure settings as identified by others (e.g. Tetley et al. for PD patients [27]). Such pathways typically identify the journey of a patient from more to less secure units, and ideally back into the community. If in the future dedicated long-stay services were developed in England, decisions would also have to be made at which point such services would become part of this pathway.

A number of authors have suggested that a lack of secure services for LD patients might contribute to their higher LoS [28] and most studies have found that severe mental illness was associated with longer and PD with shorter LoS [11]. This study did not use formal diagnostic data, but diagnostic ward type was used as a proxy and reflects these findings. It should be noted, though, that diagnostic ward type concerns whether particular groups will be admitted, not that the unit will be entirely populated by patients with that diagnosis and proportions may vary between units.

## Limitations

This was a cross-sectional study, limiting any causal inference. Lack of diagnostic data meant we had to refer to ward diagnostic classification as a proxy measure which may be unreliable. Demographic and admissions data were restricted to that which was readily obtainable within the sample. Many other variables than which were modelled in this study are likely to explain variation in long-stay.

## Conclusion

There is a large number of patients resident in English high or medium secure settings who remain in those settings for prolonged periods of time and the prevalence of long-stay varied greatly between medium secure settings suggesting potentially a lack of consistency in admission criteria and/or discharge procedures. The large number of patients admitted from the same or lower levels of security is of concern and suggests a trajectory of movement within the system rather than progression outwards. These experiences can cause a significant amount of distress for patients and carers. In order to facilitate more effective treatment and discharge of long-stay patients from secure settings, further investigation of the characteristics and needs of this patient group is required in order to identify suitable therapeutic interventions. A national strategy for the management of this patient group might assist in this.

## Footnotes

1 The different sections of the MHA differ with regards to power of decision-making regarding transfer and discharge, possibility of move back to prison, amongst other things. On the most simple level those sections designated here to the ‘civil/quasi-civil’ category allow the clinical team to decide upon the patient’s placement, while patients on restriction order require agreement by the Ministry of Justice for any transfers or discharge. Those on prison transfer orders can be moved back to prison as long as their sentence has not been served in full yet.
